# Functional Exercise Training and Undulating Periodization Enhances the Effect of Whole-Body Electromyostimulation Training on Running Performance

**DOI:** 10.3389/fphys.2018.00720

**Published:** 2018-06-13

**Authors:** Francisco J. Amaro-Gahete, Alejandro De-la-O, Guillermo Sanchez-Delgado, Lidia Robles-Gonzalez, Lucas Jurado-Fasoli, Jonatan R. Ruiz, Angel Gutiérrez

**Affiliations:** ^1^Department of Medical Physiology, School of Medicine, University of Granada, Granada, Spain; ^2^PROmoting FITness and Health Through Physical Activity Research Group, Department of Physical Education and Sports, Faculty of Sport Sciences, University of Granada, Granada, Spain

**Keywords:** WB-EMS, VO_2_max, running economy, VT2, training periodization

## Abstract

The popularity of whole-body electromyostimulation is growing during the last years, but there is a shortage of studies that evaluate its effects on physical fitness and sport performance. In this study, we compared the effects of a periodized and functional whole-body-electromyostimulation training on maximum oxygen uptake (VO_2_max), ventilatory thresholds (VT1 and VT2), running economy (RE), and lower-body muscle strength in runners, vs. a traditional whole-body-electromyostimulation training. A total of 12 male recreational runners, who had been running 2–3 times per week (90–180 min/week) for at least the previous year and had no previous experience on WB-EMS training, were enrolled in the current study. They were randomly assigned to a periodized and functional whole-body-electromyostimulation training group (PFG) (*n* = 6; 27.0 ± 7.5 years; 70.1 ± 11.1 kg; 1.75 ± 0.05 m) whose training program involved several specific exercises for runners, or a traditional whole-body-electromyostimulation training group (TG) (*n* = 6; 25.8 ± 7.4 years; 73.8 ± 9.8 kg; 1.73 ± 0.07 m), whose sessions were characterized by circuit training with 10 dynamic and general exercises without external load. The training programs consisted of one whole-body electromyostimulation session and one 20-min running session per week, during 6 weeks. The PFG followed an undulating periodization model and a selection of functional exercises, whereas the TG followed a traditional session structure used in previous studies. Both groups were instructed to stop their habitual running training program. VO_2_max, VT1, VT2, RE, and lower body muscle strength (vertical jump) were measured before and after the intervention. The PFG obtained significantly higher improvements when compared with the TG in terms of VO_2_max (2.75 ± 0.89 vs. 1.03 ± 1.01 ml/kg/min, *P* = 0.011), VT2 (2.95 ± 1.45 vs. 0.35 ± 0.85 ml/kg/min, *P* = 0.005), VO_2_max percentage at VT2 (5.13 ± 2.41 vs. 0.63 ± 1.61%), RE at VT1 (−7.70 ± 2.86 vs. −3.50 ± 2.16 ml/kg/km, *P* = 0.048), RE at 90% of VT2 (−15.38 ± 4.73 vs. −3.38 ± 4.11 ml/kg/km, *P* = 0.005), and vertical jump in Abalakov modality (2.95 ± 0.94 vs. 0.52 ± 1.49 cm, *P* = 0.008). Therefore, we conclude that running performance improvements were better after a 6-week program following an undulating periodization and consisting on functional exercises when compared with a 6-week traditional WB-EMS program.

## Introduction

Running has become increasingly popular in recent decades, and numerous novice runners start to practice this activity each year all over the world (Lee et al., 2017). Aerobic running performance is determined by different physiological parameters such as (i) maximum oxygen consumption (VO_2_max), (ii) ventilatory threshold 1 (VT1), (iii) ventilatory threshold 2 (VT2), (iv) running economy (RE) (Jones and Carter, 2000; Barnes and Kilding, 2015), and (v) muscular strength, among others (Tucker et al., 2013). Strength training is able to increase all of them (Paavolainen et al., 1999; Jones and Carter, 2000; Beattie et al., 2014). Local electromyostimulation training seems to be an alternative to traditional strength training (Filipovic et al., 2012), yet there is a lack of studies evaluating its effects on running performance-related parameters.

Whole-body electromyostimulation (WB-EMS) has recently emerged as an innovative training modality, and enables the simultaneous exogenous muscle activation of up to 18 regions with a total area of 2,800 cm^2^ covered by electrodes. It allows the configuration of different intensities in each region. To note, WB-EMS improves muscle strength in elite soccer players (Filipovic et al., 2016), in postmenopausal women (Kemmler et al., 2010), and in healthy untrained middle-aged men (Kemmler et al., 2016). WB-EMS training has some advantages: (i) training sessions are relatively short (<20 min), (ii) training frequency is low (3 sessions in 2 weeks) (Kemmler et al., 2016), (iii) sessions are dynamic and entertaining, (iv) it is a novel training stimulus, and (v) it could be used in training cessation situations such as injuries. However, to our knowledge, there are no intervention trials studying the effects of WB-EMS on aerobic performance parameters in runners.

WB-EMS intervention studies commonly use a pre-determined configuration of the electrical parameters, which is not modified during the training sessions. Moreover, the selection of exercises applied only includes motor patterns with a short range of motion and isometric actions (Kemmler et al., 2010, 2012, 2014, 2016; Kemmler and von Stengel, 2013). It is well-known that the application of a periodized training is more effective than non-periodized programs in resistance training programs (Harries et al., 2015). In addition, the application of exercise tasks involving specific running action (functional and transference task) enhance aerobic performance in endurance athletes (Balsalobre-Fernández et al., 2016). Thus, although it seems plausible that a WB-EMS training program including periodization (stimuli variation applied to achieve a specific training goal) and transference (specificity in exercise selection) as principles of sport training would result in additional benefits to those exerted by traditional WB-EMS training, empirical evidence is needed to confirm this hypothesis.

The purpose of the current study was to compare the effects of a 6-week periodized and functional WB-EMS training on VO_2_max, VT1, VT2, RE, and lower-body muscle strength in runners, vs. a traditional whole-body-electromyostimulation training. Our hypothesis is that the periodized and functional WB-EMS training will improve running performance when compared with traditional WB-EMS.

## Methods

### Participants

We recruited 15 healthy male recreational runners, who had been running 2–3 times per week (132.7 ± 28.6 min/week) for at least the previous year and had no previous experience on WB-EMS training. Three participants did not complete the study (due to job and family reasons) and were excluded from further analysis. The participants signed an informed consent. The study was approved by the Human Research Ethics Committee of the University of Granada (200/CEIH/2016) and complied with the revised ethical guidelines of the Declaration of Helsinki.

### Design

A randomized controlled trial design (ClinicalTrials.gov ID: NCT03425981) was applied following CONSORT statements. The participants were randomized into two groups: (i) the PFG (*n* = 6) and (ii) the TG (*n* = 6). Participants were instructed to do one supervised WB-EMS training session and one RE test per week, and to stop their training routines while the WB-EMS program was performed. Both WB-EMS programs sessions had the same total duration.

### WB-EMS training program

The WB-EMS training programs were designed considering different electrical parameters: (i) frequency; number of electrical pulses per time unit; it has been shown that there is not a selectively activation of muscle fibers using low or high frequencies, but fast fibers are predominantly recruited independently of the frequency applied (Gregory and Bickel, 2005), (ii) impulse width; which could influence the intensity of muscle contraction and is specific for each muscle group (Filipovic et al., 2011), (iii) impulse intensity; percentage of maximum voluntary contraction; in spite no data about it was provided in WB-EMS studies, several studies that applied local EMS showed a stimulation intensity of >50% maximum voluntary contraction is required to produce physiological improvements (Filipovic et al., 2011), and (iv) duty cycle; ratio between time receiving electrical stimuli and the total cycle time (Filipovic et al., 2011).

The WB-EMS training program consisted on six training sessions (1 per week). Before starting the program, the participants went through a familiarization session to learn movement patterns and to be adapted to the electrical stimuli. An experienced National Strength and Conditioning Association Certified Personal Trainer (NSCA-CPT) supervised all training sessions. Specific exercises of each training program modality are collected in Supplementary Material.

#### WB-EMS periodized and functional running training group (PFG)

The PFG followed an undulating periodization model (understanding periodization as a systematic planning of athletic or physical training). The training sessions were divided into four parts (the participants only did movements when receiving electrical impulse in all cases): warm up (A), strength training (B), high intensity interval power training (C), and high intensity interval training (D). The electrical parameters, except impulse intensity, were modified across different parts of the session (see Table 1) in order to follow the recommendations for each exercise modality to improve strength and aerobic performance improvements (Kemmler et al., 2014; Amaro-Gahete et al., 2017). The impulse intensity was individually adjusted by RPE every 2 min. We applied a circuit training methodology (no rest between exercises) in all phases and we did not use any external load. In phase A, the participants performed 7–10 repetitions (1 set) of 3 exercises; both concentric and eccentric phases took 2 s each in every repetition. In phase B, the participants did 1–2 sets of 5–10 repetitions of 6 exercises; the concentric phase took 1 s and the eccentric phase duration was 3 s. In phase C, the participants performed 1 set of 8 exercises; they were instructed to do as many repetitions as possible in 10 s with a 10-s rest between exercises. In phase D, the participants did 1–2 interval sets with two different intensities: moderate intensity (65% VO_2_max speed) and high intensity (>85% VO_2_max speed) running on a treadmill (30 s each set at both intensities).

**Table 1:** Electric parameters description in WB-EMS-PFG and WB-EMS-TG sessions (proposed progression).

**WB-EMS-PFG**	**Session 1**				**Session 2**				**Session 3**				**Session 4**				**Session 5**				**Session 6**			
	**W**	**S**	**HP**	**HT**	**W**	**S**	**HP**	**HT**	**W**	**S**	**HP**	**HT**	**W**	**S**	**HP**	**HT**	**W**	**S**	**HP**	**HT**	**W**	**S**	**HP**	**HT**
Total duration (min)	2	6	2	2	4	6	3	3	4	8	3	3	4	8	4	4	4	8	4	4	4	8	4	4
Frecuency (Hz)	12	55	60	20	12	65	70	25	12	75	80	35	12	85	90	40	12	85	90	40	12	85	90	40
Impulse width (μs)	350	350	350	350	350	350	350	350	350	350	350	350	350	350	350	350	350	350	350	350	350	350	350	350
RPE impulse intensity (6-20)	10	12	13	13	10	12	13	13	10	14	15	15	10	16	17	17	10	16	17	17	10	16	17	17
Duty cycle (%)	50	50	50	50	50	50	50	50	50	50	50	50	50	50	50	50	50	50	50	50	50	50	50	50
	4:4	4:4	10:10	30:30	4:4	4:4	10:10	30:30	4:4	4:4	10:10	30:30	4:4	4:4	10:10	30:30	4:4	4:4	10:10	30:30	4:4	4:4	10:10	30:30
**WB-EMS-TG**	**Session 1**				**Session 2**				**Session 3**				**Session 4**				**Session 5**				**Session 6**			
Total duration (min)	12				16				18				20				20				20			
Frecuency (Hz)	85				85				85				85				85				85			
Impulse width (μs)	350				350				350				350				350				350			
Impulse intensity (mA)	60				60				60				60				60				60			
RPE (6–20)	12–14				13–15				14–16				15–17				15–17				15–17			
Duty cycle (%)	50% (4:4)				50% (4:4)				50% (4:4)				50% (4:4)				50% (4:4)				50% (4:4)			

*WB-EMS-PFG, Whole-Body Electromyostimulation Periodized and Functional Running Training group; WB-EMS-TG, Traditional Whole-Body Electromyostimulation training group; W, Warm-up phase; S, Strength phase; HP, High Intensity Interval Power Training phase; HT, High Intensity Interval Training phase; RPE, Rated Perceived Exertion; Hz, Hertz; μs, Microseconds; mA, Milliamps*.

The exercises selected were functional and specific for the running discipline in order to comply with the principle of specificity. The exercises used in phase A (see Figure S1) were: (i) ½ squat and arm curl, (ii) ½ squat and bench flies, and (iii) ½ squat and horizontal push. The exercises used in phase B (see Figures S2.1, S2.2) were (i) lunge and knee-hip flexion, (ii) American swing, (iii) push-up, (iv) Bulgarian squat and vertical press, (v) sumo squat and lateral raises, and (vi) dead lift and horizontal pull. We used explosive and plyometric exercises in phase C (see Figures S3.1, S3.2, e.g. step jump, arms and leg frequency and cadence, climber, side step jump, skipping etc.). Finally, in phase D, the participants performed running intervals at different intensities.

The rationale of our periodization was based on the principle of progression, because it is not well-determined the best combination of electrical parameter in WB-EMS training for improve running performance.

#### Traditional WB-EMS training group (TG)

The TG program was based on training interventions applied in previous WB-EMS studies (Kemmler et al., 2010, 2012, 2014, 2016; Kemmler and von Stengel, 2013). The electrical impulse and training load were increased along the program (see Table 1). The impulse intensity was individually adjusted by RPE (Borg, 1982) every 2 min during all sessions.

The sessions were structured in a circuit format and consisted on 10 dynamic and general exercises without external load (see Figures S4.1, S4.2): (i) ½ squat and arm curl, (ii) dead lift and horizontal pull, (iii) ½ squat and trunk flexion, (v) ½ squat and vertical push, (v) ½ squat and lateral raises, (vi) ½ squat and horizontal push, (vii) squat and frontal raises, (viii) dead lift and triceps kick, (ix) squat and lateral flies, and (x) trunk rotation. The participants performed six to 10 sets (1 exercise repetition per set) depending on the total session duration, and both concentric and eccentric phases took 2 s in every repetition (a 4-s rest between exercises and 30 s between sets).

### Performance-related variables

Two assessment days took place before and after the intervention. An anthropometric evaluation and a maximal treadmill exercise test were performed on day 1, and a vertical jump test and a RE test were performed on day 2. The participants were instructed not to consume alcohol, caffeine, and not to do vigorous-intensity exercise on the 48 h prior to the assessment days. Both assessment days were separated by 48 h.

#### Anthropometry

Body mass was determined by a scale (SECA, Hamburg, Germany) and height was determined by stadiometer (SECA, Hamburg, Germany); body mass index was also calculated (kg/m^2^).

#### VO_2_max

VO_2_max was assessed in a maximum treadmill (H/P/Cosmos Pulsar treadmill, H/P/Cosmos Sport & Medical GMBH, Germany) exercise test with a progressive incremental protocol (Noakes et al., 1990; Machado et al., 2013). In brief, after a warm-up consisting on walking at 5 km/h for 3 min, the incremental protocol started with an initial speed of 8 km/h (1% grade), which was increased 1 km/h every minute until the participants reached their volitional exhaustion. Thereafter, the participants performed a cooling-down period (4 km/h and 0% grade during 5 min). O_2_ consumption and CO_2_ production were measured with a gas analyzer (Oxycon Pro; Jaeger, Höchberg, Germany). The gas analyzer was calibrated with a known gas mixture (0% O_2_ and 5.5 CO_2_) and environmental air (20.9% O_2_ and 0.03% CO_2_) immediately before each test. Consistently across assessments, the participants were strongly encouraged to invest maximum effort. The participants were previously familiarized with the 6–20 Borg scale (Borg, 1982), which was used to measure the RPE during the last 15 s of each stage and at exhaustion. Heart rate was recorded every 5 s (Polar RS300, Kempele, Finland). We also registered respiratory, RPE, and heart rate parameters during the cooling-down period. The gas exchange data was averaged every 10 s and was downloaded for later analysis.

#### Ventilatory thresholds

In the maximum treadmill exercise test, VT1 and VT2 were estimated from gas exchange data through different respiratory variables: minute ventilation (VE) and equivalents for oxygen (VE/VO_2_) and carbon dioxide (VE/VCO_2_) by two independent researchers (FA and AG), and a third researcher opinion was sought when they disagreed (AO). VT1 and VT2 were determined following a validated methodology applied in previous studies (Lucía et al., 2000).

#### Running economy

RE was determined during a treadmill protocol following a specific protocol used in previous studies (Guglielmo et al., 2009; Shaw et al., 2014; Holmes et al., 2015). The treadmill test consisted of two 10-min stages at two different intensities; (i) speed at VT1 and (ii) speed at 90% of VT2. The first 2-min record of each stage were discarded in order to ensure that the participants reached the steady-state criteria. RE (oxygen cost of running a kilometer at a specific velocity) was calculated using the following equation: RE = VO2 (ml/kg/min)/[speed (km/h)/60] (Guglielmo et al., 2009; Shaw et al., 2014; Holmes et al., 2015). RE was assessed before, during (once per week), and after the training program considering pre-intervention VT1 and 90% VT2 speed.

#### Lower body muscular strength

Vertical jump performance was assessed using the countermovement (CMJ) and Abalakov jump (ABJ) tests. In standing position, with legs straight and both hands on hips, the participant performs a vertical jump with an earlier fast counter movement. The Abalakov jump is similar to the CMJ, but now the participants is allowed to freely coordinate the arms and trunk movements to reach the maximum height (Bosco et al., 1983). The jumping height was calculated from the flight time using kinematic equations (Lehance et al., 2005) estimated by Ergo Jump Bosco System® (Globus, Treviso, Italy). Before carrying out the tests, a standardized warm-up was performed which included a 5-min run at 50% of heart rate reserve, and mobility and muscle activation exercises.

### Statistical analyses

All outcome variables were checked for normality with a graphical test (QQ-Plots) and results are expressed as mean and SD or median and ranges. The baseline and post intervention data were compared using Student's paired *t*-test. One-way analysis of covariance (ANCOVA) was used to examine the effect of group (fixed factor) on VO_2_max change, i.e., post-VO_2_max minus pre-VO_2_max (dependent variable), adjusting for baseline value. The same analyses were used for maximal aerobic speed, oxygen uptake at VT1 and VT2, VO_2_max percentage in VT1 and VT2, VT1 and VT2 speed, RE at VT1 speed and 90% of VT2 speed, and vertical jump (countermovement jump and Abalakov jump). Multiple comparisons were adjusted by Bonferroni. Two-way ANOVA (Time x Group) was used to determine changes in RE during the intervention study; we applied an adjustment by Greenhouse-Geisser when the Mauchly test of sphericity was significant (*p* < 0.05). We conducted all statistical analyses using SPSS Statistics (version 20, IBM, Ehningen, Germany) software, setting level of significance at *p* < 0.05.

## Results

There were no WB-EMS-related adverse effects. The baseline characteristics by training group are shown in Table 2. We observed no statistically significant differences at baseline (Table 3). The participants reported similar values of the impulse intensity (measured by RPE) than the pre-established ranges (mean difference ± *SD*, 0.71 ± 0.24 and 0.64 ± 0.34). This fact confirmed that participants of WB-EMS group adhered to the protocol designed in term of impulse intensity.

**Table 2:** Descriptive parameters.

	**Total (*n* = 6)**	**WB-EMS-PFG (*n* = 6)**	**WB-EMS-TG (*n* = 6)**
BODY MASS (kg)	71.9 ± 10.2	70.1 ± 11.1	73.8 ± 9.8
HEIGHT (cm)	174.7 ± 6.3	175.7 ± 5.5	173.8 ± 7.4
BMI (kg/m^2^)	23.6 ± 3	22.6 ± 2.7	24.5 ± 3.3
VO_2_max (ml/min)	3855.7 ± 615.5	3790.8 ± 812.4	3920.5 ± 404.4
VO_2_max (ml/kg/min)	53.7 ± 4.6	53.9 ± 5.3	53.5 ± 4.4
VO_2_VT1	27.7 ± 3.2	28.2 ± 3.4	27.2 ± 3.3
VO_2_ VT2	40.8 ± 4.4	40.8 ± 4.6	40.7 ± 4.5
%max VO_2_ VT1	51.6 ± 4.4	52.2 ± 2.7	51.1 ± 5.9
%max VO_2_ VT2	73.4 ± 4.8	72 ± 3.9	74.8 ± 5.4
SPEEDpeak (km/h)	16.7 ± 1.4	16.7 ± 1.6	16.7 ± 1.2
VT1s (km/h)	9.3 ± 1	9.3 ± 1.2	9.2 ± 0.8
VT2s (km/h)	13.8 ± 1.1	14 ± 1.1	13.5 ± 1
RE VT1 (ml/kg/km)	213.5 ± 12.6	210.1 ± 12	216.9 ± 13.2
RE VT2 (ml/kg/km)	246.1 ± 24.4	248.5 ± 26.3	243.7 ± 24.6
CMJ (m)	0.31 ± 0.06	0.32 ± 0.07	0.31 ± 0.05
ABJ (m)	0.36 ± 0.06	0.36 ± 0.06	0.36 ± 0.07

*BMI, Body Mass Index; VO_2_max, Maximum Oxygen Uptake; %max VO_2_ VT1, Oxygen uptake percentage in aerobic threshold relative of maximum oxygen uptake; %max VO_2_ VT2, Oxygen uptake percentage in anaerobic threshold relative of maximum oxygen uptake; VT1s, Aerobic Threshold Speed; VT2s, Anaerobic Threshold Speed; CMJ, Countermovement Jump; ABJ, Abalakov Jump; RE VT1, Running Economy Aerobic Threshold; RE VT2, Running Economy at 90% of Anaerobic Threshold; WB-EMS-PFG, Whole-Body Electromyostimulation Periodized and Functional Running Training group; WB-EMS-TG, Traditional Whole-Body Electromyostimulation training group*.

**Table 3:** Results for principal and secondary outcomes over time.

					**Test of change over time**		**Test of treatment effects**
	**WB-EMS-PFG**		**WB-EMS-TG**		**WB-EMS-PFG**	**WB-EMS-TG**	***P*****-value**
	**M**±**SD**	**CI**	**M**±**SD**	**CI**			
BODY MASS (kg)	−0.48 (1.12)	−2.66, −0.31	−0.13 (0.89)	−2.07, −0.20	**0.023**	**0.027**	0.443
BMI (kg/m^2^)	−0.52 (0.39)	−0.93, 0.07	−0.39 (0.29)	−0.62, 0.10	**0.022**	**0.021**	0.395
VO_2_max (ml/min)	104.01 (117.38)	−227.18, 19.18	21.67 (56.80)	−81.27, 37.94	0.082	0.393	0.182
VO_2_max (ml/kg/min)	2.75 (0.89)	−3.69, −1.81	1.03 (1.01)	−2.09, 0.02	<**0.001**	0.053	**0.011**
MAS (km/h)	1.01 (0.89)	−1.94, −0.06	−0.17 (0.98)	−0.87, 1.20	**0.041**	0.695	0.065
VO_2_VT1	1.43 (0.87)	−2.34, −0.51	0.67 (1.14)	−1.87, 0.53	**0.010**	0.209	0.257
VO_2_ VT2	2.95 (1.45)	−4.47, −1.42	0.35 (0.85)	−1.24, 0.55	**0.004**	0.368	**0.005**
%max VO_2_ VT1	−0.02 (1.87)	−1.94, 1.98	0.13 (2.39)	−2.63, 2.38	0.98	0.900	0.843
%max VO_2_ VT2	5.13 (2.41)	−7.66, −2.60	0.63 (1.61)	−2.32, 1.06	**0.003**	0.379	**0.008**
VT1s (km/h)	0.83 (0.75)	−1.62, −0.04	0.5 (0.55)	−1.08, 0.08	**0.042**	0.076	0.094
VT2s (km/h)	1.17 (0.41)	−1.60, −0.74	0.08 (0.38)	−0.48, 0.31	<**0.001**	0.611	<**0.001**
RE VT1 (ml/kg/km)	−7.70 (2.86)	4.70, 10.70	−3.5 (2.16)	1.24, 5.76	<**0.001**	**0.011**	0.257
RE VT2 (ml/kg/km)	5.38 (4.73)	10.42, 20.35	−3.38 (4.11)	−0.93, 7.70	<**0.001**	0.100	**0.005**
CMJ (cm)	1.47 (1.16)	−2.68, −0.25	0.70 (0.63)	−1.36, −0.04	**0.027**	**0.042**	0.203
ABJ (cm)	2.95 (0.94)	−3.93, −1.97	0.52 (1.49)	−2.08, 1.04	<**0.001**	0.434	**0.008**

*Changes in variables (Final-Baseline) for the WB-EMS-PFG and WB-EMS-TG groups over time. Change scores are Mean (SD), and confidence interval (CI). Reported p-values are for tests of an overall change over time among all subjects, and for tests of a treatment effect between MIT and HIIT groups over time. Boldface values indicate significance differences (P < 0.05). Only participants with complete intervention program were included in the complete analysis. BMI, Body Mass Index; VO_2max_, Maximum Oxygen Uptake; MAS, Maximal Aerobic Speed; VO_2_VT1, Oxygen uptake Ventilatory Threshold 1; VT2, Ventilatory Threshold 2 VT1s; %max VO_2_ VT1, Oxigen uptake at Ventilatory Threshold 1; VO_2_ VT2, Oxigen uptake at Ventilatory Threshold 2; %max VO_2_ VT1, Oxygen uptake percentage in Ventilatory Threshold 1 relative of maximum oxygen uptake; %max VO_2_ VT2, Oxygen uptake percentage in Ventilatory Threshold 2 relative of maximum oxygen uptake, VT1s, Ventilatory Threshold 1 Speed; VT2s, VT1s, Ventilatory Threshold 2 Speed; RE VT1, Running Economy at Ventilatory Threshold 1 Speed; RE VT2, Running Economy at 90% of the Ventilatory Threshold 2 Speed; CMJ, Countermovement Jump; ABJ, Abalakov Jump; WB-EMS-PFG, Whole-Body Electromyostimulation Periodized and Functional Running Training group; WB-EMS-TG, Traditional Whole-Body Electromyostimulation training group*.

### Anthropometry

BMI significantly decreased in a similar fashion in the PFG and the TG (from 22.6 ± 2.8 to 22.1 ± 2.6 kg/m^2^, and from 24.5 ± 3.3 to 24.1 ± 3.2 kg/m^2^, *P* = 0.022 and *P* = 0.021, respectively).

### VO_2_max

**Figure 1:**
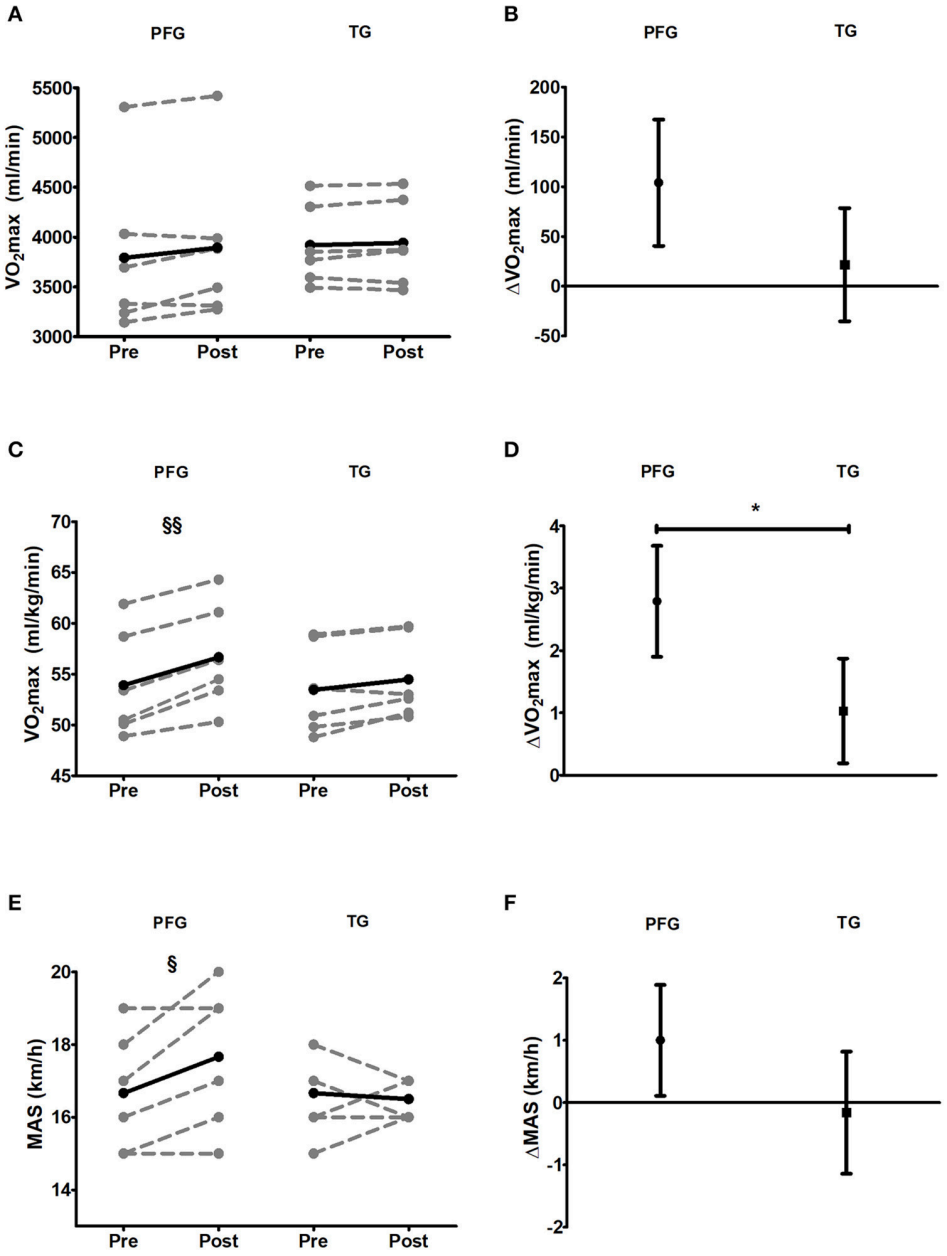
Pre and post 6-week intervention values and mean change (95% CI) in maximal oxygen uptake (absolute and relative values), and maximal aerobic speed after the intervention program. **(A,B)** Maximal oxygen uptake in absolute values [VO_2_max (ml/min)]; **(C,D)** Maximal oxygen uptake in relative values [VO_2_max (ml/kg/min)]; **(E,F)** Maximal Aerobic Speed [MAS (km/h)]. §*P* < 0.05, §§*P* < 0.01, §§§*P* < 0.001 (analysis pre-post; Student's paired *t*-test). ^*^P < 0.05, ^**^P < 0.01, ^***^P < 0.001 (analysis between groups; analysis of covariance). PFG, Periodized, and Functional group; TG, Traditional Whole-Body-Electromyostimulation training.

There were no changes in absolute VO_2_max (ml/min^−1^) levels in the TG, whereas changes were border on significance (*P* = 0.082) in the PFG (Figures 1A,B). VO_2_max in relative terms (ml/kg/min) increased in PGF group and a tendency were observed in TG after training (*P* < 0.001 and *P* = 0.053 for the PFG and the TG respectively, Figure 1C), being the pre-post changes higher in the PFG group than in the TG (mean difference: 1.72 ml/kg/min, *P* = 0.011, Figure 1D). Similarly, maximal aerobic speed increased in the PFG (*P* = 0.041, Figure 1E), whereas no changes were observed in the TG (Figure 1E). Between group changes comparisons were border of significance for maximal aerobic speed (mean difference: 1.01 vs. −0.17 km/h for the PFG and the TG, respectively, *P* = 0.065, Figure 1F).

### Ventilatory thresholds

There were no significant pre-post changes in VO2 at VT1, VO_2_max percentage in VT1, and VT1 speed in the TG (all *P* > 0.076), yet, significant pre-post differences in VO_2_ at VT1, and VT1 speed were observed in the PFG. There were no significant between groups differences when comparing pre-post changes (all *P* > 0.094, Table 3).

**Figure 2:**
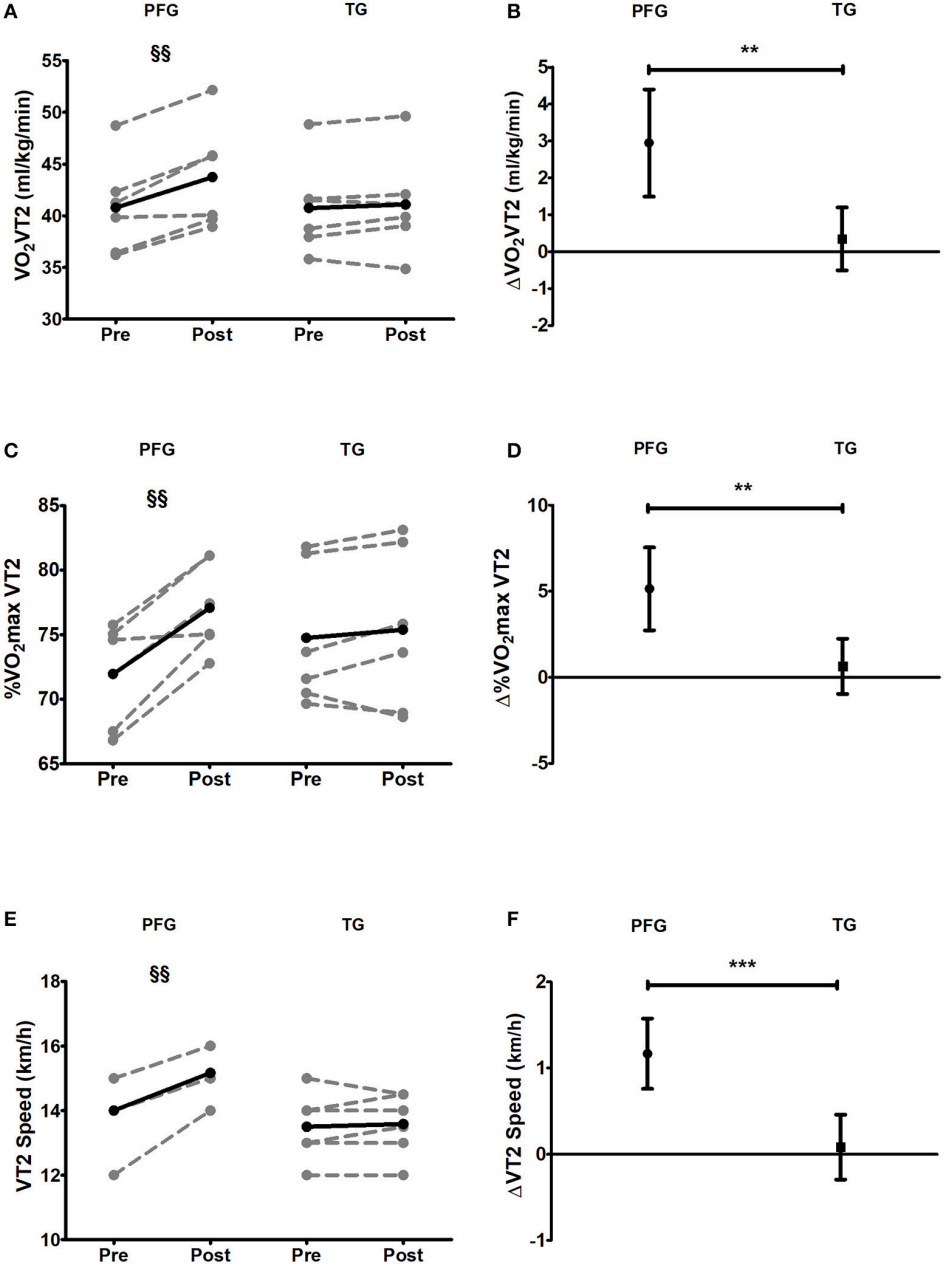
Pre and post 6-week intervention values and mean change (95% CI) in oxygen uptake at ventilatory threshold 2, maximal oxygen uptake percentage in ventilatory threshold 2, and ventilatory threshold 2 speed. **(A,B)** Oxygen uptake at ventilatory threshold 2 [VO_2_VT2s (ml/kg/min)]; **(C,D)** Maximal oxygen uptake percentage in ventilatory threshold 2 [%VO_2_max VT2]; **(E,F)** Ventilatory Threshold 2 Speed [VT2 speed (km/h)]. §*P* < 0.05, §§*P* < 0.01, §§§*P* < 0.001 (analysis pre-post; Student's paired *t*-test). ^*^P < 0.05, ^**^P < 0.01, ^***^P < 0.001 (analysis between groups; ANCOVA). PFG, Periodized and Functional Whole-Body- Electromyostimulation training; TG, Traditional Whole-Body-Electromyostimulation training.

Regarding VT2, there were significant pre-post differences in VO_2_ at VT2, VO_2_max percentage in VT2, and VT2 speed in the PFG (all *P* < 0.004), but not in the TG (all *P* > 0.360). Changes in VO_2_ at VT2, VO_2_max percentage in VT2, and VT2 speed were significantly different in the PFG and the TG (all *P* < 0.008) (see Figure 2).

### Running economy

**Figure 3:**
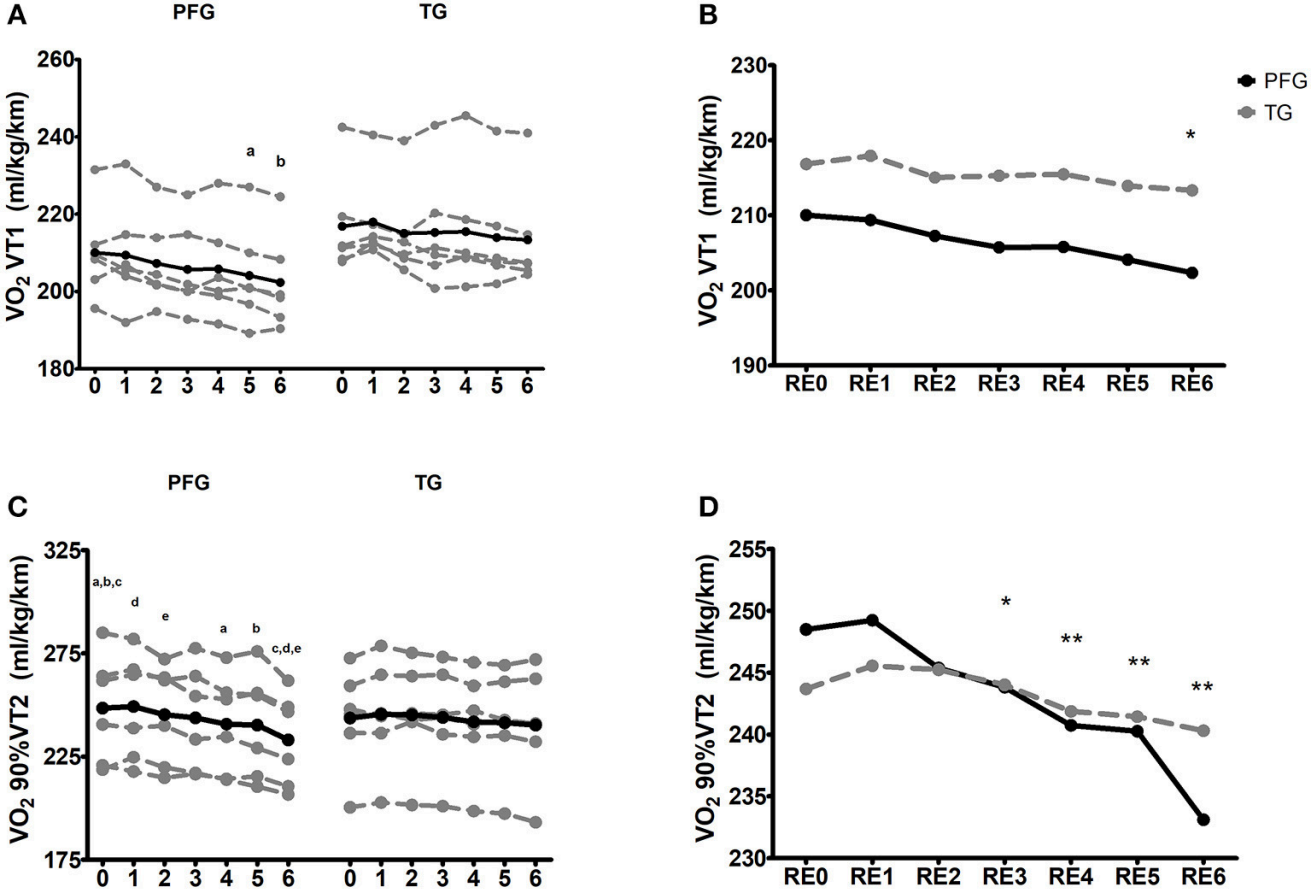
Running economy (RE) kinetics at ventilatory threshold 1 speed, and 90% of ventilatory threshold 2 speed after the intervention program. **(A,B)** Running economy at ventilatory threshold 1 speed [VO_2_ VT1 (ml/kg/km)], **(C,D)** running economy at 90% of ventilatory threshold 2 speed [VO_2_ 90%VT2 (ml/kg/km)]. RE0 corresponded to the first running economy test (pre-test); RE1 corresponded to the running economy test performed in week 1; RE2 corresponded to the running economy test performed in week 2; RE3 corresponded to the running economy test performed in week 3. RE4 corresponded to the running economy test performed in week 4; RE5 corresponded to the running economy test performed in week 5; RE6 corresponded to the running economy test performed in week 6 (post-test). Repeated letters (a–a; b–b, etc.) indicate *P* < 0.05 in different weeks (analysis throughout time; Repeated ANOVA measures). ^*^P < 0.05, ^**^P < 0.01, ^***^P < 0.001 (analysis between groups of the pre-test differences; ANCOVA). PFG, Periodized and Functional Whole-Body-Electromyostimulation training; TG, Traditional Whole-Body-Electromyostimulation training.

RE at VT1 speed and 90% VT2 speed improved in the PFG (*P* < 0.001) (Figures 3A,C, respectively), but it did not in the TG (*P* > 0.111), and significant differences were found when comparing pre-post changes between groups (*P* < 0.05 and *P* < 0.01 for VT1 speed and 90% VT2, respectively). Repeated measures analysis showed that between-group differences in RE at VT1 speed appeared after 6 weeks of intervention (*P* < 0.05), whereas differences in RE at 90% of VT2 speed appeared in the fourth week (*P* < 0.05) and continued until the post-intervention assessment (*P* < 0.01) (Figures 3B,D).

### Lower body muscle strength

**Figure 4:**
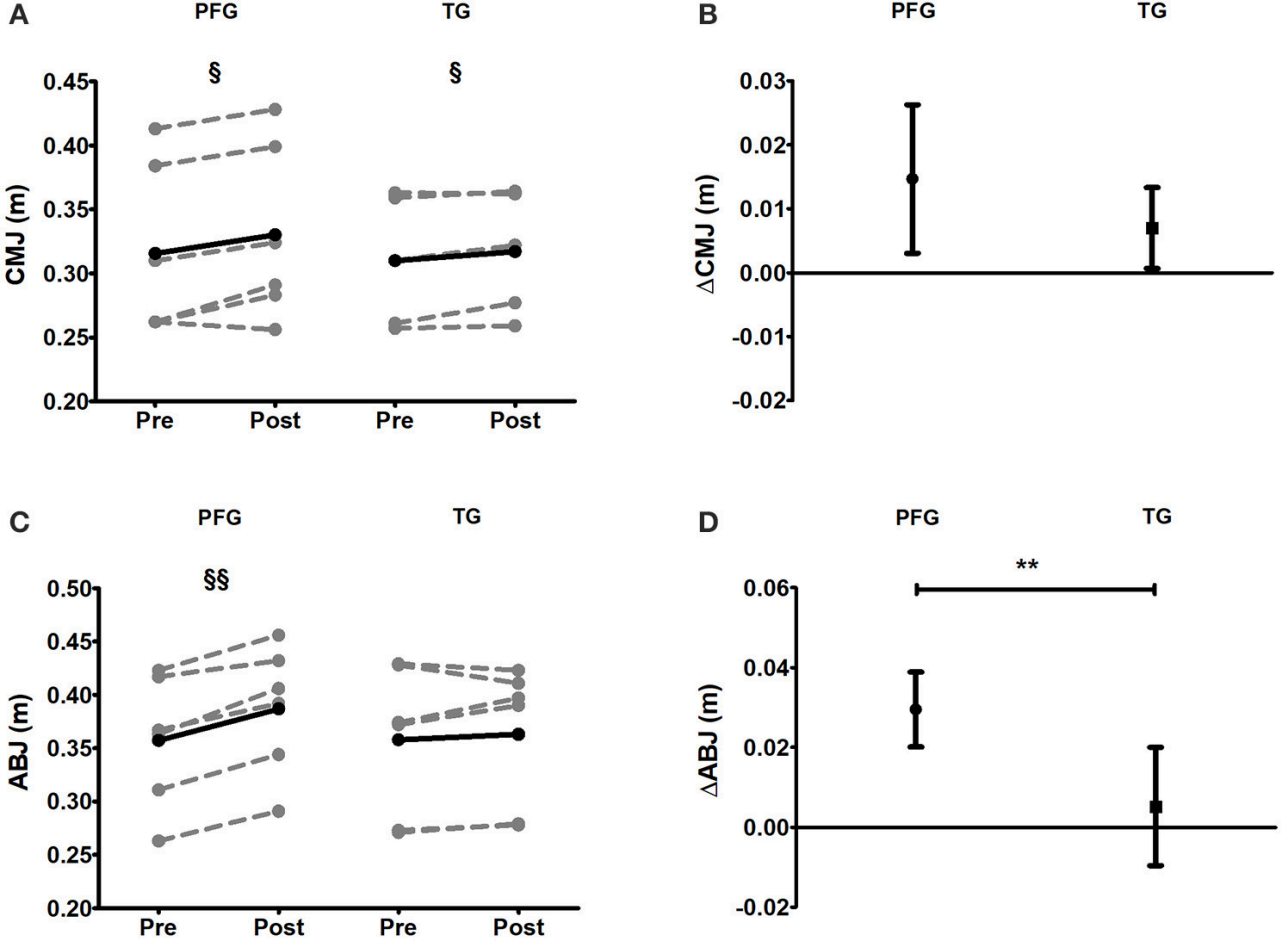
Pre and post 6-week intervention values and mean change (95% CI) countermovement jump and Abalakov jump after the intervention program. **(A,B)** Countermovement jump [CMJ (m)], **(C,D)** Abalakov jump [ABJ (m)]. §*P* < 0.05, §§*P* < 0.01, §§§*P* < 0.001 (analysis pre-post; Student's paired *t*-test). ^*^P < 0.05, ^**^P < 0.01, ^***^P < 0.001 (analysis between groups; ANCOVA). PFG, Periodized and Functional Whole-Body-Electromyostimulation training; TG, Traditional Whole-Body-Electromyostimulation training.

The levels of CMJ increased after the intervention in both the PFG and the TG (*P* < 0.05 in both cases), being pre-post changes similar between groups (*P* = 0.203) (Figures 4A,B). The pre-post changes in ABJ were, however, different between the PFG vs. the TG (*P* < 0.01, Figures 4C,D), and we observed no pre-post differences in the TG (*P* = 0.724).

## Discussion

This study shows that the effects of a 6-week periodized and functional WB-EMS training program on running performance-related parameters including VO_2_max, VT2, RE, and ABJ in recreational runners are better than those observed after following a traditional WB-EMS training program. These findings reinforce the fact that the effects of WB-EMS training on running performance can be greatly improved if it is correctly designed. To our knowledge, this is the first study analyzing the effect of WB-EMS on running performance-related parameters in recreational runners.

### VO_2_max

VO_2_max is considered one of the best predictor of running performance (Tucker et al., 2013). We observed an increment of 2.75 ml/kg/min in the PFG. These findings do not concur with an intervention program consisting on two combined strength sessions per week which lasted for 11 weeks while maintaining its endurance training volume in trained athletes, which increased several strength parameters and muscle fiber cross sectional area of both fiber type I and fiber type II, but no changes in VO_2_max were found (Vikmoen et al., 2016). However, a meta-analysis (Sloth et al., 2013) evaluated the effects of high intensity interval training interventions (lasting 2–8 weeks) on VO_2_max in healthy sedentary or recreationally active adults, and showed a similar VO_2_max increase (ranged between 4.2 and 13.4%). These improvements are similar to those observed in the PFG.

Traditionally, WB-EMS training programs do not vary electrical frequency, electrical intensity, width impulse, and duty cycle, within the training sessions. Moreover, the selection of exercises usually consists on a set of non-functional movements (i.e., not motor related with the competition movement) (Filipovic et al., 2011; Amaro-Gahete et al., 2017). We designed a training intervention that followed (i) an undulating periodization training model, and (ii) a functional selection of exercises to improve running performance. We observed that absolute VO_2_max increased by 2.78% after the 6-week intervention program in the PFG compared with the TG. When expressed in relation to body mass (ml/kg/min), an improvement on VO_2_max could be explained either by a reduction on body mass and/or an increase in VO_2_max itself (ml/min). Our data suggest that improvements obtained in VO_2_max relative to body mass were determined only by body mass loss in the TG; however, VO_2_max in relative terms were strongly influenced by VO_2_max in absolute terms (*P* = 0.08) in the PFG.

Periodized resistance training programs result on higher benefits over muscle strength compared to non-periodized training programs, which also seems to be the case in WB-EMS training programs (Williams et al., 2017). On the other hand, Nuhr et al. reported that a local electromyostimulation training program (10 weeks, 4 h per day, 7 days per week) at low frequency (15 Hz) applied in quadriceps and hamstring muscles of both legs in healthy men improve maximal aerobic-oxidative capacity (Nuhr et al., 2003). Interpreting our results, despite the fact that both WB-EMS training programs had the same time exposure (same number of sessions and duration), we cannot discard that differences in overall training load (e.g., different muscular tension) explain the observed differences.

### Ventilatory thresholds

It is well-known that VT1 and VT2, especially the latter, have an important role in long-distance running performance (Jones and Carter, 2000; Barnes and Kilding, 2015). The effect of concurrent training on VT1 and VT2 is controversial. Two studies reported little changes on VT2 in runners (Mikkola et al., 2011; Støren et al., 2013), which concur with our findings in the TG. In contrast, others observed substantial improvements in VT2 (Guglielmo et al., 2009; Taipale et al., 2013) after following resistance training program in trained athletes. In our study, the PFG experienced an increase of 7% in VO_2_ at VT2. Of note is that an improvement in VT1 and VT2 speed, and VO_2_ at VT1 and VT2, could be an indirect effect of an improvement on VO_2_max and/or RE (see below). However, the increase of VO_2_max percentage at which VT2 is achieved suggest that a direct improvement of VT2 was observed in the PFG.

### Running economy

RE, defined as the oxygen consumption required at a given absolute submaximal exercise intensity, is considered critical for running performance in trained individuals with homogenous VO_2_max (Saunders et al., 2004). Our findings concur with those of Taipale et al. who reported substantial improvements in RE after 8 weeks of combined explosive strength and endurance training in runners (Taipale et al., 2013).

Although we cannot know whether the periodization or the use of functional exercises are the main cause of the improvement in RE, the performance of functional exercises with running actions at maximal speed with superimposed electromyostimulation could be an important factor to improve RE. The PFG included a period of high intensity short run intervals with superimposed electromyostimulation; this fact could determine some advantages over the TG inasmuch this training modality did not include running actions. Therefore, this fact could contribute to maximize performance at high speed intensity. On the other hand, we cannot discard that following a periodization training model could increase the improvements obtained (Harries et al., 2015).

### Lower body muscle strength

Several studies have shown the importance of lower body muscle strength in running performance, especially in RE (Dumke et al., 2010). Our findings concur with those observed by other studies using WB-EMS and local electromyostimulation training (12–28 sessions) on the lower body muscles in trained athletes (Babault et al., 2007; Filipovic et al., 2011, 2016). Filipovic et al. (2016) applied a WB-EMS intervention twice a week, which consisted on 3 × 10 maximal squat jumps with a set pause of 60 s. They found significant increases in squat jump, CMJ, and drop jump (all *P* < 0.05), which concur with our findings. Our study showed an increase in CMJ in both training groups, whereas significant improvement in ABJ was only observed in the PFG. This fact could be explained by differences in the exercises used and the electrical parameters applied in both intervention groups (e.g., the inclusion of several plyometric exercises in the PFG). Since the main difference between CMJ and ABJ is the contribution of interlimb coordination, the improvement obtained in ABJ in the PFG when compared to the TG, suggest that a periodized and functional WB-EMS training program could have a considerable impact on inter-limb coordination (Dal Pupo et al., 2013). However, more studies are needed to elucidate which physiological or biomechanical mechanism may explain the differences reported between groups.

Possible reasons which could explain the physiological improvements observed are: (i) a better efficiency of mitochondrial metabolism, what is related with an increase in the rate of lipid utilization [it is well-known that this fact produces an important descent in rate of glycogen depletion (Esfarjani and Laursen, 2007)], (ii) an increased capillary density after an specific training program, yet we applied low frequencies in different parts of PFG sessions and it is well-known that low frequencies produce a development of capillarization process (Miyamoto et al., 2016), and (iii) positive changes in neurological process related to lower limb coordination, recruitment and synchronization of motor units, and co-activation of muscles, which could results in a greater leg stiffness and muscular strength (Barnes and Kilding, 2015); only PFT include an wide values spectrum of the different electrical parameters manipulated, and specific functional task (plyometric or explosive strength exercises) oriented to improve these facts. All of the previous items could produce a delay in local and peripheral fatigue resulting in a decrease of oxygen uptake for a specific speed and, consequently, getting a better running performance.

### Limitations

The results of this study should be considered with caution as there are some limitations. The main limitation of this study (as all WB-EMS previous studies) is the lack of information about the total electrical impulse magnitude applied. Indeed, the best methodology to control WB-EMS training load is currently unknown. Moreover, the low sample size and the inexperienced participants in WB-EMS training may have influenced the results, since improvements produced by the application of a novel stimulus are expected. In addition, we cannot know whether these results extend to longer training programs, and or to not professional athletes or patients. We did not confirm VO_2_peak by checking VO_2_max plateau with a constant work rate test (110% of the maximal intensity reached after the ramp protocol test) because our participants were recreational runners and this validation is not necessary in this population (Poole and Jones, 2017). One of the strengths is that we included a positive control group which allowed to avoid the existence of false positive cases.

## Conclusion

A 6-week periodized and functional WB-EMS training program produces considerable higher on VO_2_max, VT2, RE, and ABJ in recreational runners compared with a traditional WB-EMS training program. Our findings indicate that WB-EMS training, as a novel stimulus, could complement or even modify the common training methods in runners. In addition, our results strongly support that WB-EMS training must be carefully designed and supervised, not only for safety reasons, but also for efficiency issues. This concept is particularly relevant in the commercial context where this type of technology could be understood as a completely autonomous training methodology. More studies are needed to better understand the practical applications of this new training methodology in other populations, to elucidate whether the improvements obtained are due to the exercises selected, or to the electrical parameters, or to its combination, and also to elucidate whether a traditional strength program get higher improvements in running performance than WB-EMS training.

## Data availability statement

The raw data supporting the conclusions of this manuscript will be made available by the authors, without undue reservation, to any qualified researcher.

## Author contributions

FA-G, AD-l-O, LR-G, and AG conceived and designed research. FA-G, AD-l-O, LR-G, and LJ-F conducted experiments. FA-G and JR analyzed data. FA-G wrote the manuscript. AG and JR revised the manuscript. All authors read and approved the manuscript.

### Conflict of interest statement

The authors declare that the research was conducted in the absence of any commercial or financial relationships that could be construed as a potential conflict of interest.
