# Supplementary material for: Functional Exercise Training and Undulating Periodization Enhances the Effect of Whole-Body Electromyostimulation Training on Running Performance
Source: Front Physiol. 2018 Jun 13;9:720. doi: 10.3389/fphys.2018.00720 (PMC6009337; doi:10.3389/fphys.2018.00720)
Supplement: Supplementary file 1 [file Image_1.PDF]

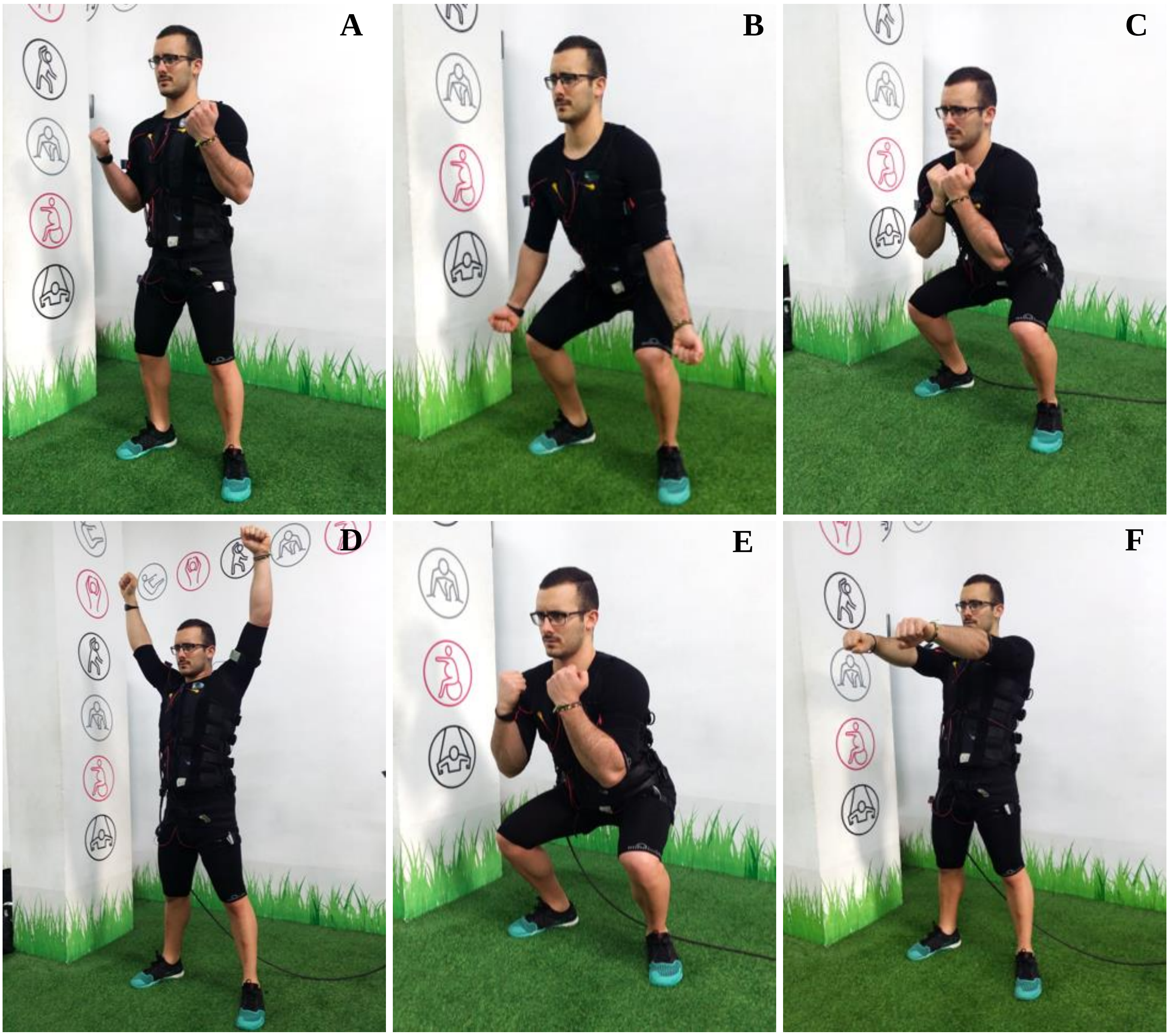

Figure S1: Exercises used in whole-body electromyostimulation training program functional and periodized in warm-up section. A and B*;* ½ squat and arm curl. C and D*;* ½ squat and bench flies. E and F*;* ½ squat and horizontal push.

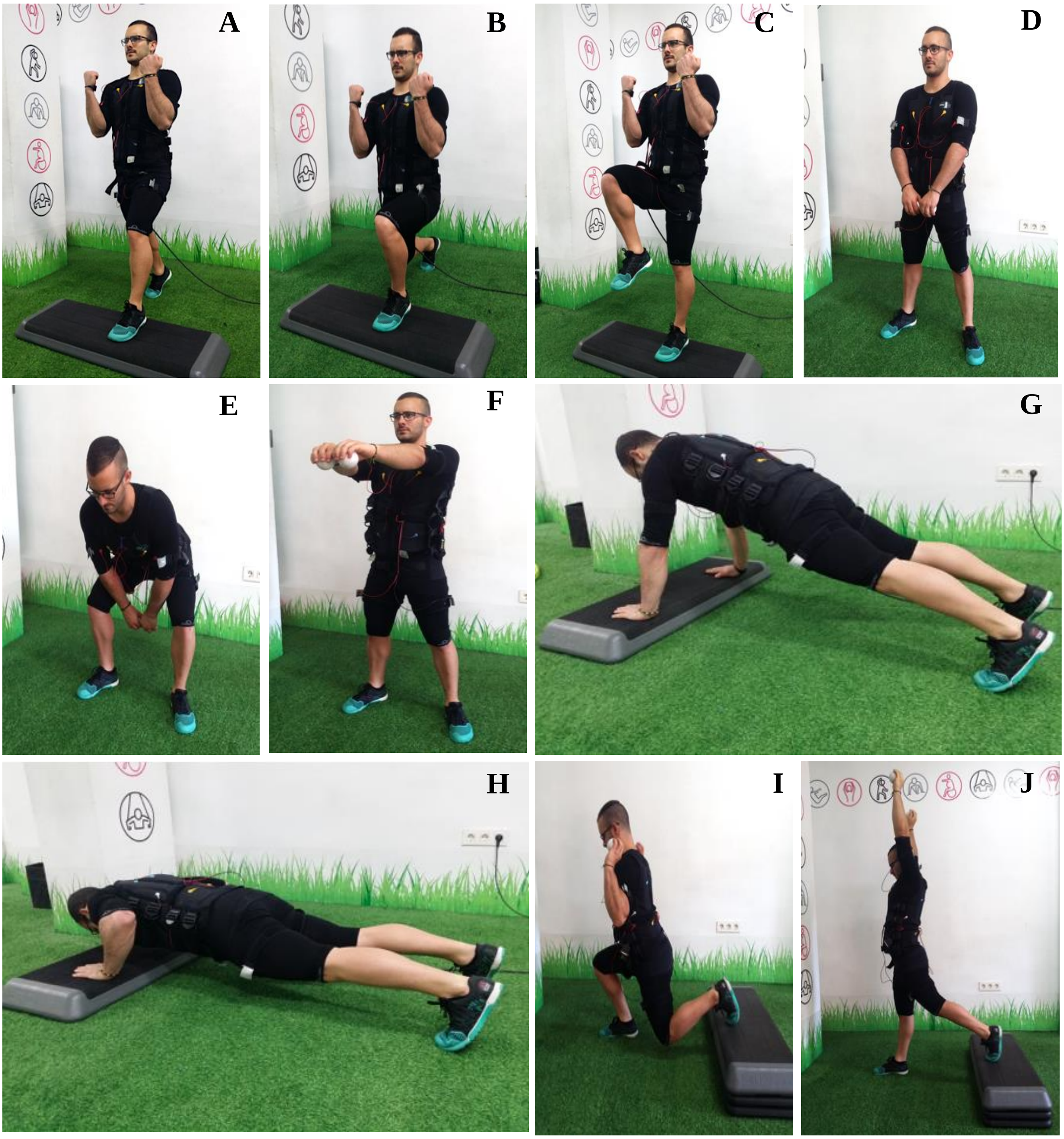

Figure S2.1: Exercises used in whole-body electromyostimulation training program functional and periodized in strength section. A, B and C; lunge and knee-hip flexion. D, E and F; American swing. G and H; push-up. I and J; Bulgarian squat and vertical press.

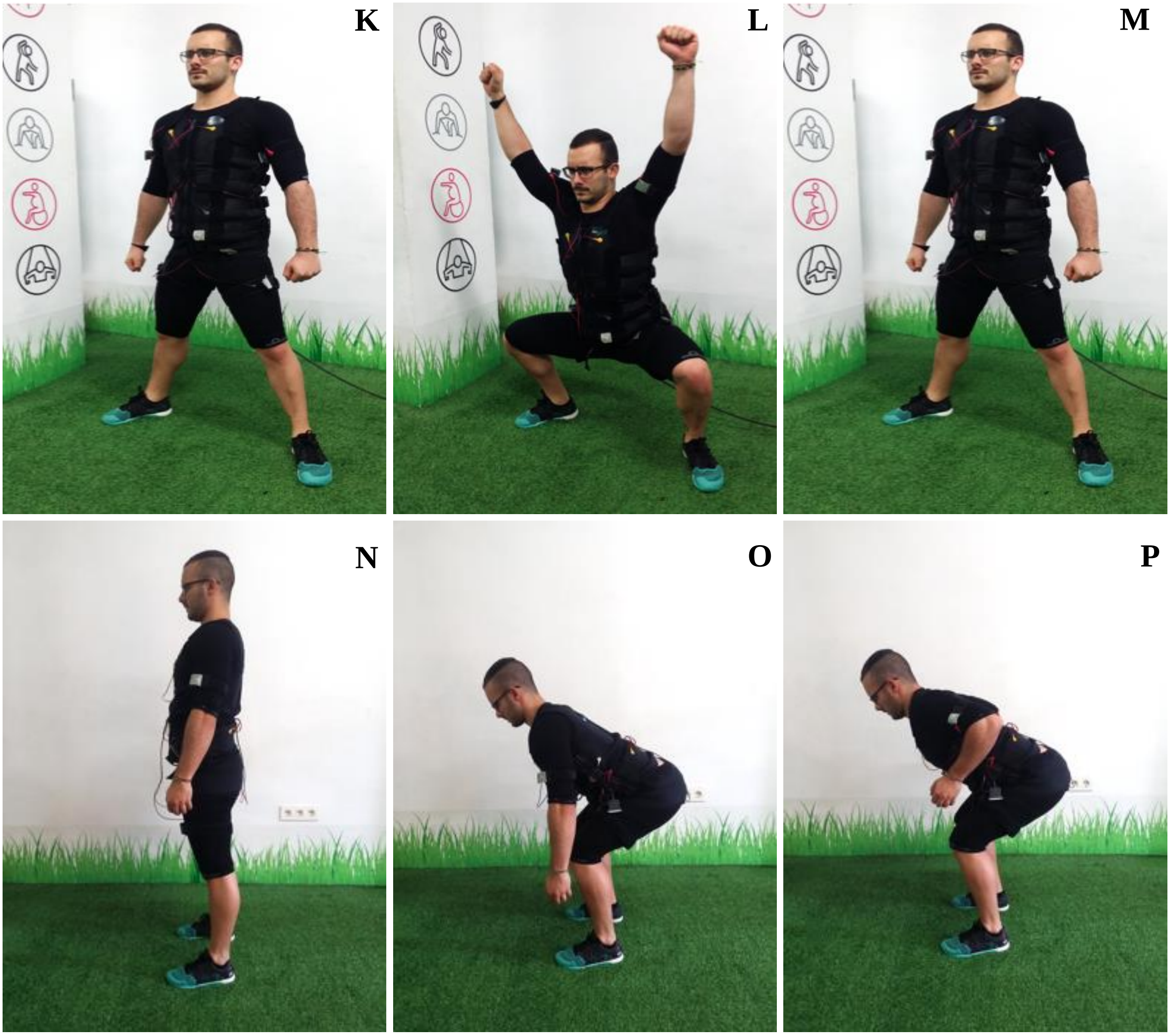

Figure S2.2: Exercises used in whole-body electromyostimulation training program functional and periodized in strength section. K, L and N; sumo squat and lateral raises. N, O and P; dead lift and horizontal pull

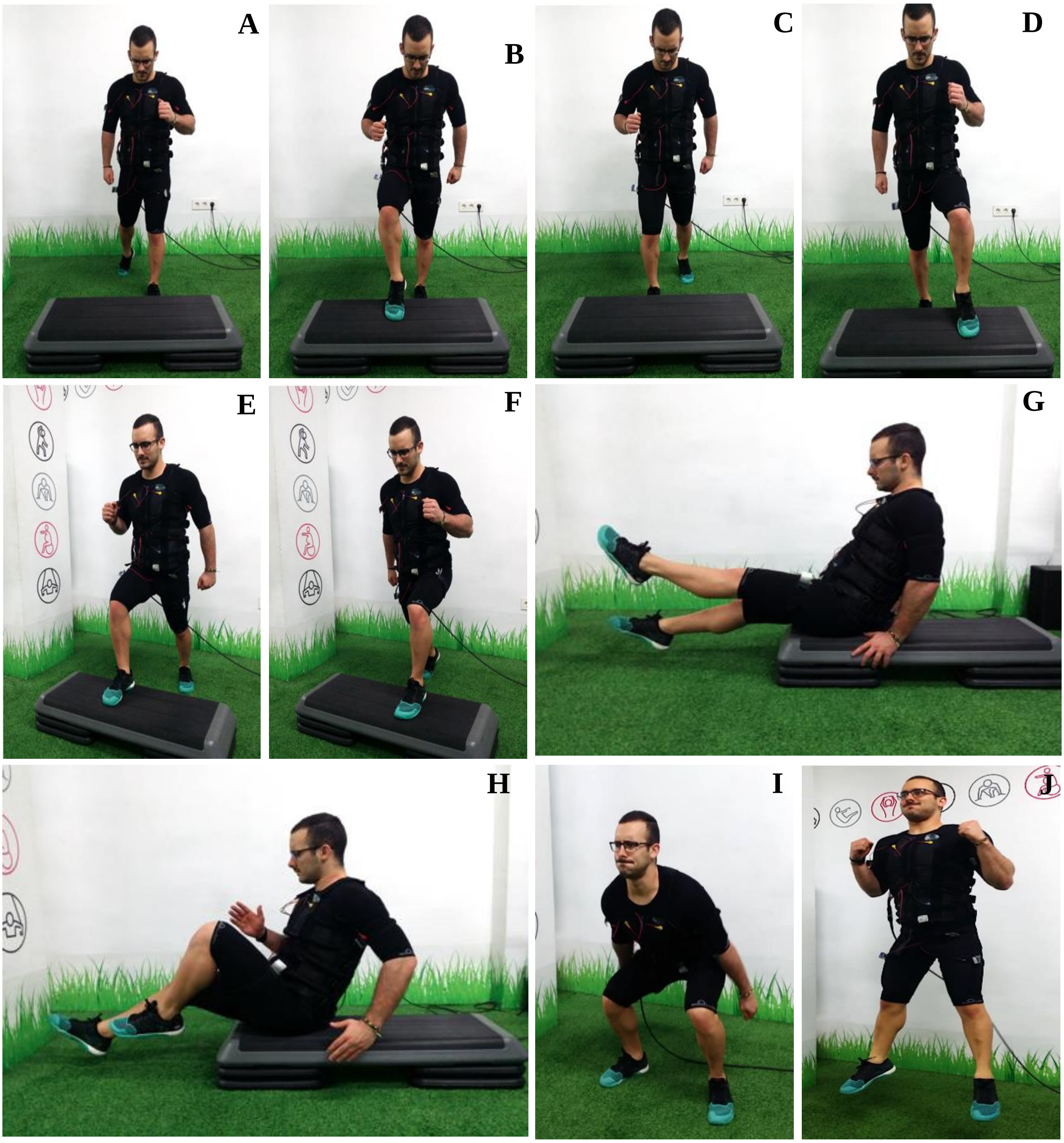

Figure S3.1. Exercises used in whole-body electromyostimulation training program functional and periodized in high intensity interval power training section. A and B; right leg frequency and cadence. C and D; left leg frequency and cadence. E and F; step jump. G and H; heels frequency and cadence (sitting). I and J; arms frequency and cadence (sitting).

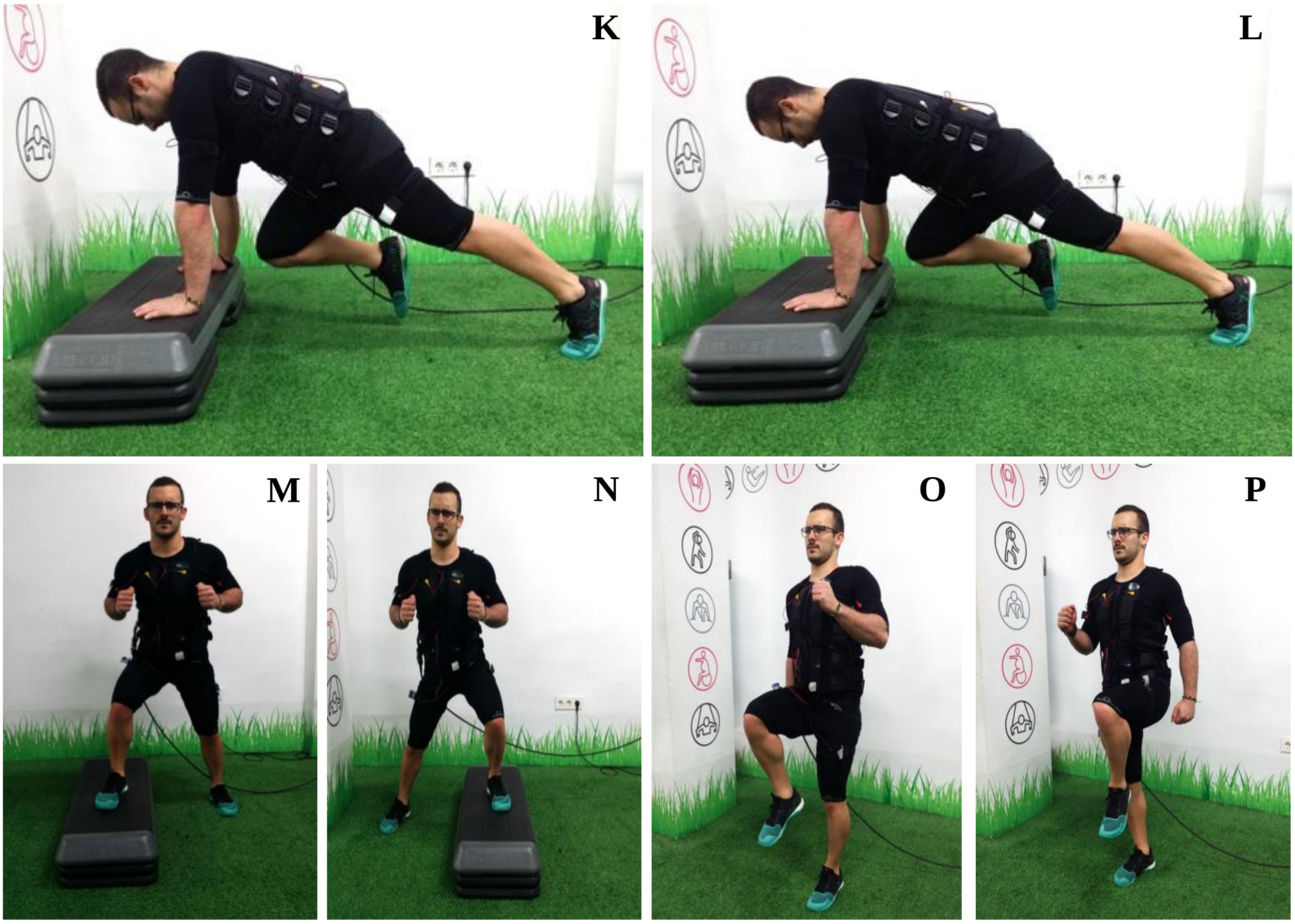

Figure S3.2. Exercises used in whole-body electromyostimulation training program functional and periodized in high intensity interval power training section. K and L; climber. M and N; side step jump. O and P; step jump.

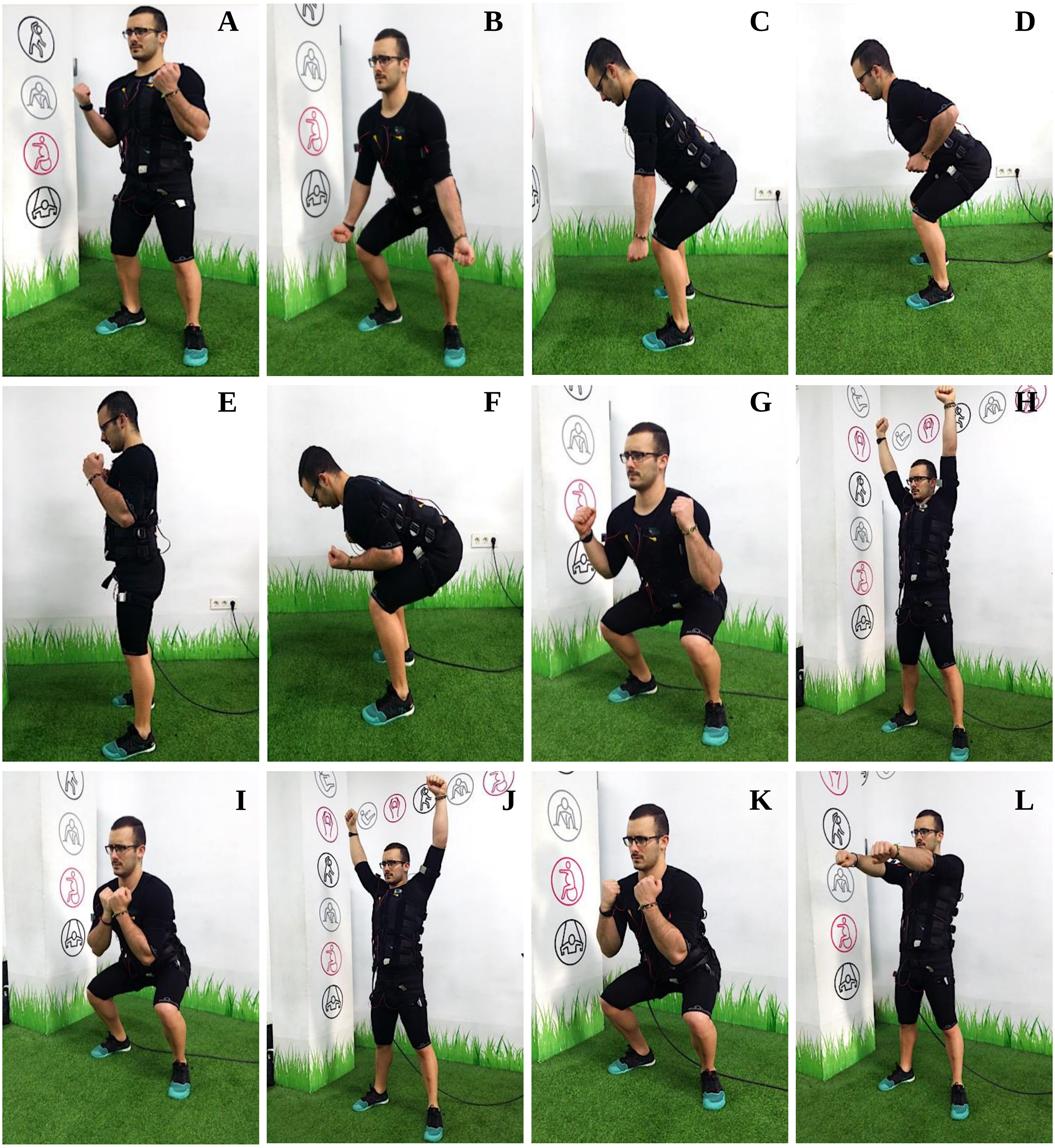

Figure S4.1. Exercises used in traditional whole-body electromyostimulation training program (non-functional and non-periodized). A and B; ½ squat and arm curl. C and D; dead lift and horizontal pull. E and F; ½ squat and trunk flexion. G and H; ½ squat and vertical push. I and J; ½ squat and lateral raises. K and L; ½ squat and horizontal push.

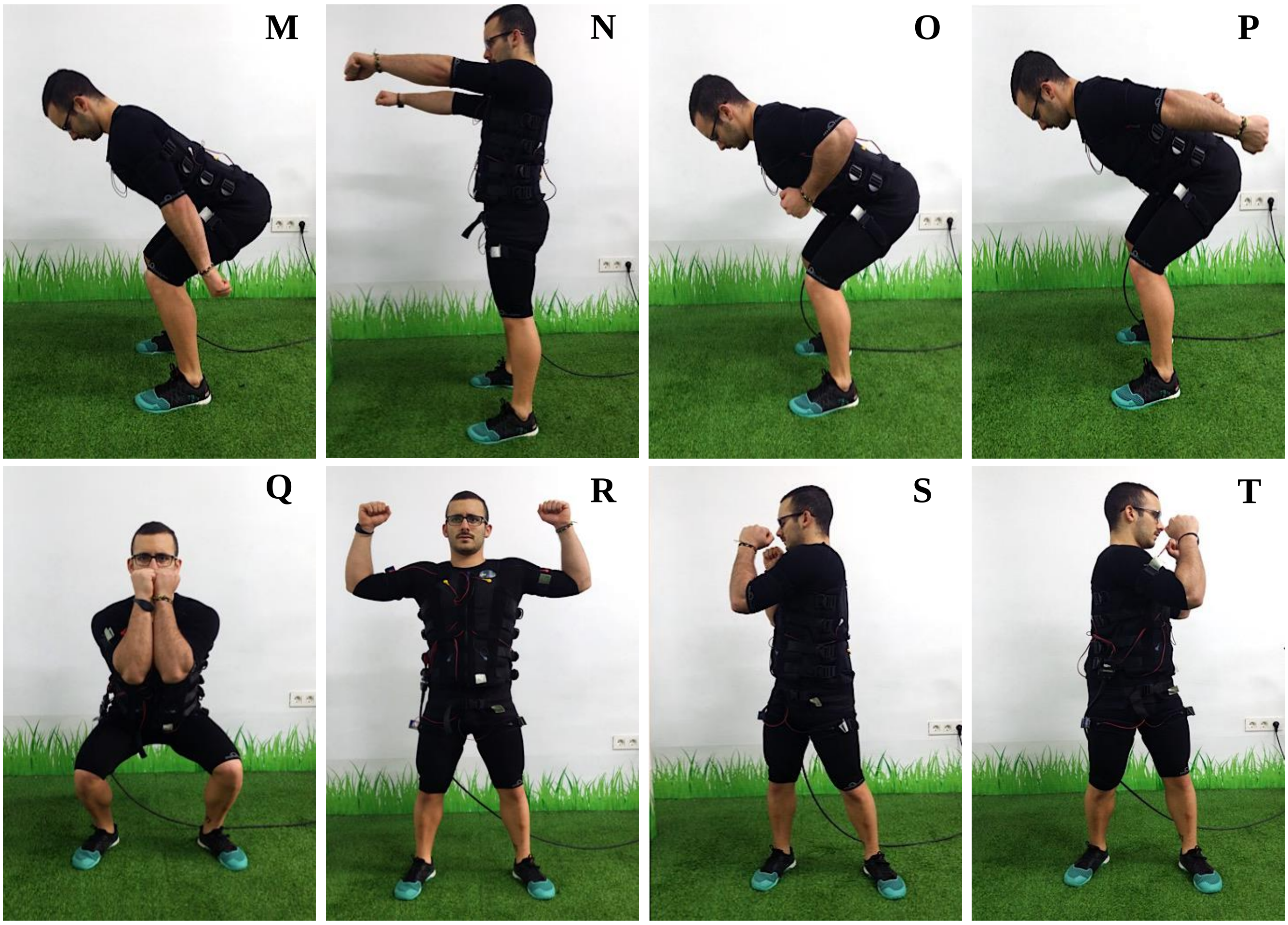

Figure S4.2. Exercises used in traditional whole-body electromyostimulation training program (non-functional and non-periodized. M and N; ½ squat and frontal raises. O and P; dead lift and triceps kick. Q and R; ½ squat and lateral flies. S and T; trunk rotation.
